# Cross-Sectional Analysis of Serologic Response to Arthropod-Borne and Hemorrhagic Fever Viruses in Ghanaian Livestock Herders

**DOI:** 10.4269/ajtmh.25-0452

**Published:** 2026-05-05

**Authors:** Keersten Ricks, Stephanie Monticelli, Seth Offei Addo, Tamara Clements, Mba-Tihssommah Mosore, Ronald E. Bentil, Janice Tagoe, Clara Yeboah, Eric Behene, William Asiedu, Daniel Mingle, Sandra Abankwa Kwarteng, Dorcas Atibilla, Victor Asoala, Christopher Stefan, Andrew Herbert, Terrel Sanders, Anne T. Fox, Samuel K. Dadzie, Andrew G. Letizia, Randal Schoepp, Shirley C. Nimo-Paintsil

**Affiliations:** ^1^ Diagnostic Systems Division, U.S. Army Medical Research Institute of Infectious Diseases, Fort Detrick, Maryland;; ^2^ Virology Division, U.S. Army Medical Research Institute of Infectious Diseases, Fort Detrick, Maryland;; ^3^ The Geneva Foundation, Tacoma, Washington;; ^4^ Parasitology Department, Noguchi Memorial Institute for Medical Research, University of Ghana, Legon, Ghana;; ^5^ Public Health Division, Ghana Armed Forces, Accra, Ghana;; ^6^ Department of Theoretical and Applied Biology, College of Science, Kwame Nkrumah University of Science and Technology, Kumasi, Ghana;; ^7^ Kintampo Health Research Center, Bono East Region, Kintampo, Ghana;; ^8^ Navrongo Health Research Center, Upper East Region, Navrongo, Ghana;; ^9^ U.S. Naval Medical Research Unit—European, Africa, and Central Commands (EURAFCENT), Ghana Detachment, Accra, Ghana

## Abstract

Zoonotic diseases account for more than 60% of emerging infectious diseases, and they are the leading cause of pandemics. As humans and livestock become increasingly transient and the environment and climate change, disease vectors expand into previously untouched geographical regions, spreading pathogens. In this study, we assessed the seroprevalence of arthropod-borne and hemorrhagic fever viruses of similar clinical presentation and endemicity in high-risk populations in Ghana (animal handlers). Using a microneutralization assay, we compared total IgG prevalence with live virus neutralizing response. In total, 300 blood samples were collected from consenting healthy adults at five military and three civilian sites across Ghana. The observed seroprevalence rates for Rift Valley fever virus (RVFV), Crimean–Congo hemorrhagic fever virus (CCHFV), Ebola virus, Lassa virus, and Marburg virus were 14.7%, 7.0%, 2.3%, 1%, and 0%, respectively. Microneutralization data further verified virus-specific neutralization positives of the total IgG positives. Among animal handlers who had recently skinned livestock, 19 (25.3%) were exposed to RVFV, and 20 (28.6%) of those in the coastal savannah ecological zone were also more likely to be exposed to RVFV compared with those in the other ecological zones (*P* = 0.002). Animal handlers younger than 25 years old had a higher exposure rate to CCHFV than those older than 25 years old (*P* <0.001). These data help us better understand the risk of exposure to zoonotic and vector-borne diseases in the region. Moreover, this study establishes methods for assessing seropositivity in a multiplexed format for higher-throughput sample analysis.

## INTRODUCTION

The human–animal interface has been studied for decades in an attempt to understand how pathogens spill over from animal hosts to human populations that live and work in these environments.^1^ Zoonotic diseases are characterized by pathogenic organisms that can be shared naturally between vertebrate animals and humans.^2^ It is estimated that more than 60% of emerging infectious diseases are caused by zoonoses.^3^ As human populations expand into wildlife habitats, livestock movement increases, international travel escalates, and urbanization continues to spread, the risk of spillover increases. The Ebola virus (EBOV) outbreaks in West and Central Africa reinvigorated discussions on zoonotic spillover, pandemic preparedness, and rapid response to “Disease X.” However, the entire world faced a challenge when the coronavirus disease 2019 pandemic made large-scale spillover a reality and exposed critical gaps in the global pandemic response.^4^^,^^5^ Researchers moved at a rapid pace to fill these gaps with the development of diagnostics, vaccines, and therapeutics.^6^^,^^7^

Diagnostic assays, whether molecular or immunologic, are key to understanding disease pathogenesis, detecting infected individuals, tracking disease spread, and evaluating prevalence in the community—be it humans, vertebrate animals, or vectors. Real-time polymerase chain reaction is used for both pathogen detection (qualitative) and determining viral load (quantitative). Pathogen-specific IgM and IgG antibody immunological assays characterize the infection-specific host response and diagnostically, are commonly used to screen for recent or previous pathogen exposure. Integrating both molecular and serologic methods for biosurveillance adds confidence to these findings. Medical countermeasures, such as vaccines and therapeutics, have been largely at the forefront of scientific and media discussions as a touchstone to a prepandemic society. Furthermore, diagnostics and disease surveillance methods that are accessible to endemic regions are critical to the predictive modeling of disease emergence and spread.^8^

The Diagnostic Systems Division at the U.S. Army Medical Research Institute of Infectious Diseases (USAMRIID) has had a long-standing collaboration with military and civilian partners to build capacity in overseas laboratories for integrated disease biosurveillance.^9^^–^^11^ We recently partnered with our collaborators at the Noguchi Memorial Institute for Medical Research of the College of Health Sciences at the University of Ghana together with our U.S. Naval Medical Research Unit—EURAFCENT, Ghana Detachment to retrospectively analyze a cohort of livestock ranchers and abattoir workers for serologic evidence of exposure to Crimean–Congo hemorrhagic fever virus (CCHFV) and other zoonotic diseases known to be endemic to West Africa. A report in 2021 lists the majority of these diseases as viral zoonoses of national importance in Ghana.^12^ Given the occupational risk of exposure to ticks, mosquitoes, and contaminated bodily fluids, the likelihood of observing serologic response to zoonotic pathogens is expected to be higher for this study population. Utilizing magnetic bead-based multiplexed serologic assays developed on the Luminex Magpix system (Luminex, Austin, TX), we screened the serum samples for IgG prevalence to CCHFV as well as Rift Valley fever virus (RVFV), EBOV, Lassa virus (LASV), Marburg virus (MARV), alphaviruses, and flaviviruses. Additionally, we assessed whether samples neutralized live virus to further characterize the serologic response.

## MATERIALS AND METHODS

### Human samples.

**Figure 1.**
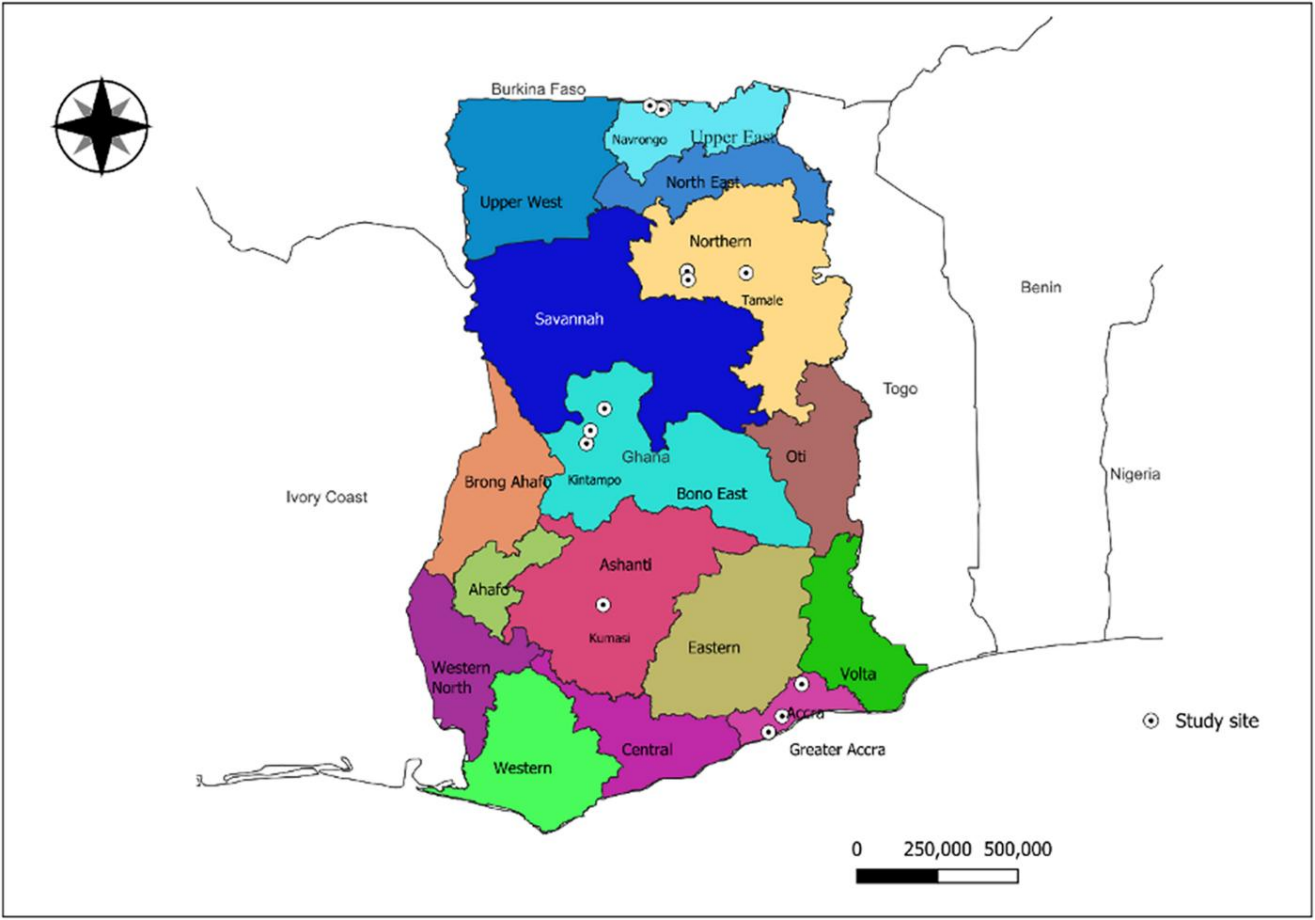
Map of Ghana showing study enrollment sites. The map was developed using Quantum Geographical Information System v. 3.36.3.

Healthy livestock ranchers/abattoir workers who had contact with live animals and/or animal parts were eligible for participation. The study was carried out between January and September 2020. The study population comprised individuals 18 years of age and older who were animal handlers, ranchers, or abattoir workers and raised livestock within the following study sites: Accra, Kumasi, Kintampo, Tamale, and Navrongo (Figure 1). The majority of the population was expected to be males; however, females were enrolled when they met the inclusion criteria. A required sample size of 300 individuals (rounded up from the calculated number of 288) was calculated. Sample size calculations were made with the following assumptions. Population size (estimated number of livestock keepers/handlers in the study areas based upon local veterinarian estimate) was 6,724 people; anticipated human seroprevalence rate was 26.7% for tick-borne infections^13^ with a 5% margin of error and a 95% confidence level.

Blood samples (10 mL) were collected from the enrolled participants and placed in serum separator tubes. These samples were centrifuged in the field, and the serum was aliquoted. The whole-blood and serum samples were then transported at −20°C to the U.S. Naval Medical Research Unit—EURAFCENT, Ghana Detachment laboratory located at the Noguchi Memorial Institute for Medical Research. A questionnaire was administered to each participant that captured the demographics, occupational exposure, clinical signs, and symptoms within the last year.

### Magnetic bead multiplex serology immunoassay.

To assess seroprevalence to high-risk pathogens in Ghanaian livestock workers, we created a multiplex of five zoonotic viruses (CCHFV, LASV, RVFV, MARV, and EBOV) and two pan viral family assays (pan alphavirus and pan flavivirus) using the Luminex Magpix platform (Luminex, Austin, TX). Briefly, serum samples were diluted at 1:100 in phosphate-buffered saline with 0.05% Tween-20 (PBST; Sigma-Aldrich, St. Louis, MO) and phosphate-buffered saline with 0.05% Tween-20 and 5% skim milk (PBST-SK). Recombinant proteins were coupled to individually addressable Magplex microspheres using the Luminex xMAP^®^ antibody coupling kit according to the manufacturer’s instructions. The complete list of proteins and bead regions is detailed in Supplemental Table 1. Each antigen-coupled bead was mixed at a 1:1 ratio before diluting in PBST to 5 × 10^4^ microspheres/mL and added to the wells of a Costar (Corning, Corning, NY) polystyrene 96-well plate at 50 *µ*L per well (2,500 microspheres of each antigen bead set per well). The plate was placed on a magnetic plate separator (Luminex) covered with foil, and microspheres were allowed to collect for 60 seconds. While still attached to the magnet, the buffer was removed from the plate by inverting and disposing into a sink. Then, 50 *µ*L of the diluted plasma samples were added to appropriate wells. The plate was covered with a black vinyl plate seal and incubated with shaking at 400 rpm on a microplate shaker for 1 hour at room temperature (RT). Using the plate magnet to retain the Magplex microspheres in the wells, the plate was washed three times with 100 *µ*L of PBST for each wash. Fifty microliters of a 1:100 dilution of goat anti-human IgG phycoerythrin conjugate (P9170, Millipore Sigma, Burlington, MA) in PBST-SK was added to each well. The plate was covered again with a black vinyl plate seal and incubated with shaking for 1 hour at RT. After incubation, the plate was washed three times as detailed above, and the Magplex microspheres were resuspended in 100 *µ*L of PBST for analysis using the Magpix multiplex assay. All samples were run in duplicate. Raw data were reported as median fluorescence intensity for each bead set in the multiplex. The raw signal was normalized against a known negative American pooled human serum sample to calculate the signal-to-noise (S/N) value. Samples with an S/N value greater than 20 were considered positive. For serologic analyses of diagnostically unknown populations at a single collection point, a “4-fold” increase over baseline is not stringent enough to establish a positivity rate because of variations in a true population baseline. This baseline is challenging to establish for true unknown populations for seven different viruses. An S/N cutoff of 20 closely mimics a limit of quantitation calculation (α_negative_ + 10 × SD_negative_) from a distribution of normal American serum for each target but normalizes each assay for ease of interpretation relative to known positive controls.

### Rift Valley fever virus MP12 plaque reduction neutralization test assay.

Aliquots of human serum samples were shipped to the Diagnostic Systems Division, USAMRIID (Fort Detrick, MD) for the plaque reduction neutralization test (PRNT) analysis. The PRNTs were conducted on selected sera as previously described.^14^ Briefly, serum samples were heat inactivated for 30 minutes at 56°C and diluted 4-fold from 1:10 to 1:10,240 in Hank’s balanced salt solution containing penicillin/streptomycin and 5% heat-inactivated fetal bovine serum. Diluted serum samples were tested for their ability to neutralize approximately 100 plaque forming units of the challenge virus. Each sample dilution was tested in duplicate. Both known positive and negative control sera were included with every assay. Serum–virus mixtures were incubated overnight at 4°C and then inoculated onto 90–95% confluent monolayers of the appropriate cell lines grown in six-well tissue culture plates. After incubation for 1 hour at 37°C, a nutrient-rich 0.6% agarose overlay was added, and plates were incubated at 37°C for the appropriate number of days for the virus. Plates were then stained with a second overlay containing 5% neutral red, and plaques were counted 24–48 hours later. Titers were recorded as the reciprocal of the highest serum dilution reducing 80% of the plaque assay dose, and a probit titer was calculated using the forecast function in Microsoft Excel (Microsoft Corp, Redmond, WA). A probit titer was determined using an equation representing the average number of plaques counted per well as well as the corresponding dilution for each serum sample and then forecasting the exact dilution that would correspond to the number of plaques used as the 80% cutoff. The PRNT virus strain used was RVFV MP12.

### Microneutralization assay.

The microneutralization analysis was conducted in the Virology Division, USAMRIID. Specimens were heat inactivated in a 56°C water bath for 30 minutes to inactivate complement. Heat-inactivated serum specimens were diluted 1:10 in cell culture media (minimum essential media [MEM]; 10-010, Corning) containing 2% heat-inactivated fetal bovine serum (GE Healthcare Hyclone, Chicago, IL), and three-log dilutions were performed in duplicate. Plasma from naive individuals and plasma from convalescent individuals were used as negative and positive controls, respectively. Diluted serum was mixed with MARV-Ci67, LASV-Josiah, EBOV-Zaire’95, or CCHFV-IbAr10200; incubated at 37°C for 1 hour; and added to Vero E6 cells at a target multiplicity of infection of 0.2, 0.75, 1, or 0.5, respectively. Unbound virus was removed after a 1-hour incubation at 37°C. Cells were washed once in Dulbecco’s phosphate-buffered saline without calcium and magnesium (DPBS; Millipore Sigma), and culture media (MEM + 5% fetal bovine serum + 1% penicillin-streptomycin; 15140122, Gibco Thermo Fisher Scientific, Waltham, MA) was added 24 hours postinfection. Cells were fixed in 10% formalin for 24 hours, washed three times with DPBS, permeabilized with 1% Triton X-100 (BIO-RAD, Hercules, CA), and blocked with Cell Staining Buffer (BioLegend, San Diego, CA). The number of infected cells was determined using antibodies specific for each of the viruses: anti-mouse CCHFV nucleoprotein-specific monoclonal antibody (mAb) 9D5, anti-human EBOV glycoprotein-specific mAb KZ52, anti-mouse LASV glycoprotein-specific mAb L52161-6, or anti-human ebolavirus glycoprotein-specific mAb ADI-15742 (MARV) followed by goat anti-mouse or anti-human crossadsorbed IgG (H&L) Alexa Fluor 488 fluorescently labeled secondary antibody (A-11001, Invitrogen Thermo Fisher Scientific, Waltham, MA) and NucBlue Live ReadyProbes Reagent (33342, Hoechst, Thermo-Fisher, Eugene, OR). The percentage of infected cells was determined with a Cytation5 plate reader and Gen5.11 software (BioTek, Santa Clara, CA). Percentage neutralization for each serum sample at each dilution was determined relative to untreated, virus-only control wells; values were log_10_ normalized, and then, the area under the curve was calculated and plotted utilizing the baseline percentage of infection of a naïve human sera sample. Samples were determined to be positive if greater than the log of the area under the curve of the naive human sera sample. All Biosafety level4 (BSL4) virus manipulation and neutralization work was conducted in a BSL4 environment following all institutional biosafety standard operating procedures and regulatory requirements.

### Neutralization of live virus.

After assessing the seropositivity rate using the Magpix multiplex, we determined whether cohorts of the Magpix-positive samples neutralized live virus when compared with negative serum samples. It is known that not all antibodies are neutralizing, but neutralization assays can serve as a layered in vivo method to assess functional antibody titer.^14^ We used a traditional PRNT to screen samples for RVFV neutralization activity at BSL2 using the MP12 RVFV strain. The higher-throughput microneutralization assays for the BSL4 pathogens (EBOV, MARV, CCHFV, and LASV) were used because of the space, time, and reagent constraints of working in BSL4 containment suites. Given that the highest seroprevalence observed outside of the alphaviruses and flaviviruses was against RVFV, we explored live virus neutralization with all samples with an S/N value greater than 10 and 30 samples with an S/N value less than 10.

## RESULTS

### Study participant demographics.

Of the 300 livestock handlers, 69% were older than 35 years of age, with the majority of the total participants being males (87.7%) (Table 1). Per the responses from the questionnaire that was administered to each participant, 84% and 63.7% of these had been exposed to caring for live animals and handling of animal parts, respectively, within the last 3 months before the study commenced. An additional 51% of the participants cared for animals outside of their routine work (Supplemental Tables 2a, 2b, 3a, and 3b).

**Table 1:** Study participants’ demographic characteristics associated with seroprevalence of infection: pan alphavirus, pan flavivirus, and Rift Valley fever virus

Characteristics	*N* (%)	Pan Alphavirus			Pan Flavivirus			RVFV		
		*n* (%)	95% CI	*P*-Value*	*n* (%)	95% CI	*P*-Value*	*n* (%)	95% CI	*P*-Value^†^
Sex										
Male	263 (87.7)	164 (62.4)	56.3–68.0	0.539	202 (76.8)	71.3–81.5	0.34	43 (16.4)	12.3–21.4	0.028^‡^
Female	37 (12.3)	25 (67.6)	50.8–80.8		31 (83.8)	68.0–92.6		1 (2.7)	0.4–17.4	
Age (years)										
<25	28 (9.3)	11 (39.3)	23.0–58.4	0.009^‡^	21 (75)	55.6–87.8	0.293	3 (10.7)	3.4–29.0	0.002^‡^
25–34	65 (21.7)	38 (58.5)	46.1–69.9		51 (78.5)	66.7–86.9		5 (7.7)	3.3–17.4	
35–44	83 (27.7)	51 (61.5)	50.5–71.4		59 (71.1)	60.4–79.9		6 (7.2)	2.8–15.3	
>44	124 (41.3)	89 (71.8)	63.2–79.0		102 (82.3)	74.4–88.1		30 (24.2)	17.4–32.6	
Region										
Greater Accra	60 (20.0)	31 (51.7)	39.0–64.1	<0.001^‡^	55 (91.7)	81.3–96.5		20 (28.6)	19.1–40.3	0.002^‡^
Ashanti	70 (23.3)	30 (42.9)	31.7–54.8		28 (40.0)	29.1–51.9	<0.001^‡^	2 (4.0)	1.0–1.5	
Brong Ahafo	50 (16.7)	36 (72.0)	57.9–82.8		43 (86.0)	73.2–96.5		7 (11.7)	5.6–22.7	
Northern	60 (20.0)	35 (58.3)	45.4–70.2		47 (78.3)	66.0–87.1		5 (8.3)	3.5–18.7	
Upper east	60 (20.0)	57 (95.0)	85.4–98.4		60 (100)	–		10 (16.7)	9.1–28.5	
Total	300	189 (63.0)	57.4–68.3		233 (77.7)	72.6–82.0		44 (14.7)	11.1–19.2	

RVFV = Rift Valley fever virus.

* The *P*-value was obtained using the χ^2^ test.

^†^ The *P*-value was obtained using the Fisher exact test.

^‡^ Statistical significance at *P* <0.05.

### Total IgG prevalence using Magpix multiplexed serology.

**Figure 2.**
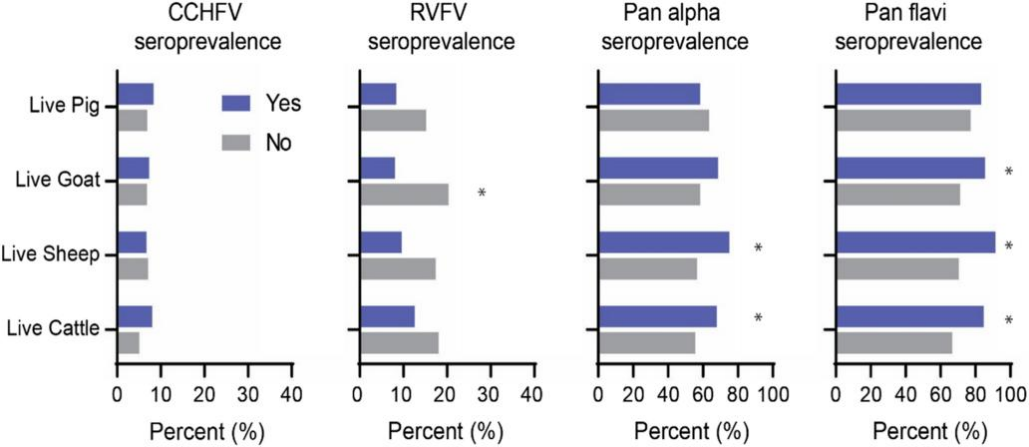
Occupational exposure risks to febrile and vector-borne diseases. CCHFV = Crimean–Congo hemorrhagic fever virus; RVFV = Rift Valley fever virus. Asterisks represent statistical significance at *P* <0.05. *p*-value was obtained for the pan assays using chi-square test. *p*-values for CCHFV and RVFV were obtained using fisher's exact test.

Seroprevalence of CCHFV, LASV, RVFV, MARV, EBOV, filoviruses, and alphaviruses was assessed utilizing a multiplex Magpix assay. Seroprevalence rates recorded for the samples tested were as follows: flaviviruses: 77.7% (*n* = 233/300); alphaviruses: 63.0% (*n* = 189/300); RVFV: 14.7% (*n* = 44/300); CCHFV: 7.0% (*n* = 21/300); EBOV: 2.3% (*n* = 7/300); and LASV: 0.3% (*n* = 1/300). Marburg virus, on the other hand, recorded zero seroprevalence (Tables 1 and 2). A scatterplot of the S/N values of all samples is shown in Supplemental Figure 1. There was significant risks of exposure to alphaviruses among animal handlers who worked on live cattle and sheep: 67.8% and 75%, respectively. Similarly, flavivirus seroprevalence was significantly greater among animal handlers working on live cattle (*n* = 155/183; 84.7%), sheep (*n* = 95/104; 91.4%), and goats (*n* = 117/134; 85.4%) (Figure 2). Again, handlers of slaughtered goat parts were at a greater risk of being exposed to pan flavivirus (*n* = 46/49; 93.9%). Also, livestock herders who had recently cared for live animals were highly exposed to pan flavivirus (*n* = 207/252; 82.1%) (Supplemental Tables 4a and 4b). Those who reported having fever and weight loss were mostly exposed to pan alphavirus (Supplemental Tables 5a and 5b) Generally, there was a high seroprevalence of these zoonotic viruses in the northern part of Ghana compared with the south. The greater Accra region (*n* = 20/60; 28.6%), which is in the coastal part of Ghana, recorded the most exposure to RVFV. The seroprevalence of EBOV was 2.3%, and it was detected among animal handlers who had recently cared for live animals and those who handled slaughtered animal parts. Seroprevalence of CCHFV was 7%, and it was mostly detected among those who had recently cared for live animals (Tables 1 and 2).

**Table 2:** Study participants’ demographic characteristics associated with seroprevalence of infection: Ebola virus and Crimean–Congo hemorrhagic fever virus

Characteristics	*N* (%)	EBOV			CCHFV		
		*n* (%)	95% CI	*P*-Value*	*n* (%)	95% CI	*P*-Value*
Sex							
Male	263 (87.7)	6 (2.3)	1.0–5.0	1.000	16 (6.1)	3.7–9.7	0.157
Female	37 (12.3)	1 (2.7)	0.4–17.4		5 (13.5)	5.6–29.0	
Age (years)							
<25	28 (9.3)	1 (3.6)	0.5–22.2	0.894	8 (28.6)	14.7–48.1	0.001^†^
25–34	65 (21.7)	1 (1.5)	0.2–10.3		3 (4.6)	1.5–13.5	
35–44	83 (27.7)	2 (2.4)	0.6–9.2		4 (4.8)	1.8–12.2	
>44	124 (41.3)	3 (2.4)	0.8–7.3		6 (4.8)	2.2–10.4	
Region							
Greater Accra	60 (20.0)	1 (1.7)	2.2–11.1	0.073	5 (8.3)	3.5–18.7	0.132
Ashanti	70 (23.3)	0 (0.0)	–		1 (2.9)	0.7–10.9	
Brong Ahafo	50 (16.7)	0 (0.0)	–		1 (2.0)	0.3–13.2	
Northern	60 (20.0)	2 (3.3)	0.8–12.5		7 (11.7)	5.6–22.7	
Upper east	60 (20.0)	4 (6.7)	2.5–16.6		6 (10.0)	4.5–20.7	
Total	300	7 (2.3)	1.1–4.8		21 (7.0)	4.6–10.5	

CCHFV = Crimean–Congo Hemorrhagic fever virus; EBOV = Ebola virus.

* The *P*-value was obtained using the Fisher exact test.

^†^ Statistical significance at *P* <0.05.

Frequency distributions of the viral targets data can be seen in Supplemental Figure 2. The distribution of values for the pan alphavirus and pan flavivirus assays is much wider than for the other assays given the endemicity of those arboviruses. This is expected as alphaviruses, such as Chikungunya (CHIKV) and endemic viral diseases such as Dengue (DENV), are endemic to the region, and there are yellow fever vaccination campaigns that would cause the pan flavivirus IgG assay to be positive.

### Live virus neutralization.

We observed significant neutralization at the PRNT50 of live RVFV in samples with an S/N greater than 40 (Supplemental Figure 4). Low-level neutralization was observed in samples with an S/N between 20 and 40. Any samples with an S/N less than 20 were determined to have no virus neutralization. No real neutralization trend was observed for CCHFV by microneutralization assay, even though we recorded 7% total antibody seroprevalence by Magpix; interestingly, the one sample with an S/N greater than 40 for LASV neutralized live virus. Minimal neutralization was observed for a few MARV and EBOV samples tested (Supplemental Figure 3).

## DISCUSSION

In this study, we used a multiplexed magnetic bead-based immunoassay platform, the Luminex Magpix, to screen Ghanaian livestock workers for antibody prevalence to zoonotic viral pathogens, including filoviruses (EBOV and MARV), arenaviruses (LASV), bunyaviruses (CCHFV and RVFV), flaviviruses, and alphaviruses. This cohort of samples was of interest because the participants live and work directly at the human–animal interface, introducing inherent occupational risk of exposure to zoonotic diseases. Serological assays are crucial for surveillance because they record immunological history and exposure patterns, even while diagnostics that can identify active infections are essential for controlling outbreaks. The Luminex Magpix platform’s multiplexed serology allowed for effective screening for numerous viruses and produced results that were in line with other seroprevalence investigations in comparable populations. Additionally, we observed a high seroprevalence of the zoonotic viruses in the northern part of Ghana compared with in the south. This can be attributed to the high livestock dependence of inhabitants, resulting in close human–animal contact, which facilitates infections.^15^^,^^16^ Furthermore, the environment of northern Ghana coupled with the trade and movement of humans with their livestock across the Burkina Faso border into Ghana contributes to the risk of infections.^17^ Of the viral hemorrhagic fevers screened, the highest IgG prevalence rates were found for CCHFV and RVFV at 7.0% and 14.7%, respectively. Rift Valley fever virus is a mosquito-borne virus mostly seen in domesticated animals, but it has been known to spill over into humans who come in close contact with infected animals. Neighboring countries have reported RVFV outbreaks, but Ghana has not yet identified active cases. Notably, ecological and epidemiological variables may be responsible for the increased seroprevalence of RVFV seen in the population from Accra. Accra is a significant metropolitan and periurban area with opportunities for humans to interact with infected animals and mosquito vectors. Recent data from southern Ghana indicate RVFV circulation among herdsmen (17.8% in herders) and their flocks (overall prevalence of 13.7%) using RVFV-specific ELISAs.^18^ The findings from this study underscore the importance of continuous One Health surveillance and combined vector and animal reservoir control measures in this area. Although active cases of CCHFV, a tick-borne virus, in Ghana have not been described, CCHFV has been detected in Ghana in ticks collected off of infected cattle,^16^^,^^19^^,^^20^ and a whole-genome sequence of CCHFV was generated from an infected tick in the northeast region of Ghana.^20^ Furthermore, a 2016 study of abattoir workers in Kumasi indicated a 5.7% seroprevalence to CCHFV using a commercial CCHFV ELISA.^19^ The absence of an association between CCHFV IgG detection and live virus neutralization may be partially explained by decreasing immunity; nonetheless, studies conducted in Uganda have revealed persistent IgG responses in survivors.^21^ Crossreactivity of the Magpix CCHFV ELISA with antibodies against related orthonairoviruses, including Nairobi sheep disease virus, that are probably circulating but unidentified in the area is another conceivable explanation.^22^ Although nucleoprotein-based assays offer superior specificity, glycoprotein-targeting ELISAs frequently crossreact with closely related viruses.^22^ In regions with complex viral landscapes, virus neutralization assays are crucial for improving seroprevalence interpretations, and they are still the gold standard for verifying particular exposure.

Low seroprevalence rates were observed for EBOV, MARV, and LASV. Although Ghana is geographically close to Liberia, Guinea, and Sierra Leone, the three countries that were devastated by the EBOV outbreak in 2014–2016, there were no reported cases of EBOV in Ghana at that time. However, our assay detected antibodies in 2.3% of the samples tested. There is reported evidence of antinucleoprotein EBOV antibodies in fruit bats in Ghana, but there is no reported serosurveillance data from human samples in the country.^23^ Another study reported low seroprevalence to anti-EBOV glycoprotein in Ghanaian pigs.^24^ No seropositivity to MARV was noted in this cohort; however, given the three MARV cases in 2022 in the Ashanti region, continued prospective surveillance for MARV exposure is incredibly important.^25^ Only one sample was IgG positive for LASV. The first documented cases of LASV infection in Ghana were reported in 2011 in the Ashanti region.^26^ Both cases shared risk factors of hunting and livestock interaction in the same community, but the patients themselves had no interaction. More recently, there was an outbreak of LASV in the greater Accra region in early 2023 with 27 confirmed cases and one death.^27^

Antibody prevalence rates were highest in the alphavirus and flavivirus families at 63.0% and 77.7%, respectively. By using virus-like particles expressing the full-envelope proteins of CHIKV and DENV to capture circulating IgG, we knowingly chose to use a crossreactive approach to IgG detection for these two arboviral families as these pathogens are known to be endemic to the region.^28^^,^^29^ In places, like Ghana, where vaccination against yellow fever is a common public health measure, it is crucial to interpret flavivirus pan IgG serological data cautiously. Strong and durable antibody responses are produced by the yellow fever vaccine, which has been widely used in childhood immunization programs and mass campaigns over the past few decades. In serological tests, these vaccine-induced antibodies may crossreact with other flaviviruses, thus producing false-positive results or overestimating the population’s natural exposure to flaviviruses. It is difficult to distinguish between antibodies produced by vaccination and those resulting from spontaneous infections in the absence of comprehensive vaccination histories of study participants. This restriction encourages the adoption of complementary diagnostic methods to improve specificity, and it highlights the necessity of carefully contextualizing seroepidemiological data in relation to existing public health measures.

This study emphasizes the value of One Health strategies in viral zoonosis surveillance, integrating environmental, animal, and human data through cooperative collaborations. In resource-constrained environments, the multiplexed serological approach presented here provides a scalable platform that can be adjusted for integrated surveillance to guide focused treatments and lower disease risk in susceptible groups.

The study could not perform neutralization assays on the potential alphavirus and flavivirus candidates in an attempt to identify virus-specific neutralization activity as we were limited in sample volume to screen them. However, this would be an interesting future study on a subset of the positives to understand what specific viruses are circulating in these regions. The presence of neutralizing antibodies is thought to constitute a major component of the acquired immunity to the virus.^30^ Neutralization assays are also commonly used as a secondary assay to bolster seroprevalence studies. Although we saw a good correlation between RVFV and LASV IgG seroprevalence and live virus neutralization, there was no real correlation for CCHFV. As we have no information regarding when these individuals were infected, neutralizing antibodies may wane over time postexposure.

## CONCLUSION

Data from this study help to better understand the risk of exposure to CCHFV and other zoonotic/vector-borne diseases in the region. They also establish methods for assessing seropositivity in a multiplexed format for higher-throughput sample analysis. Although the seroprevalence rates for the hemorrhagic fever viruses were low overall, increased surveillance, access to surveillance tools, and education on safe animal-handling practices can aid in reducing the risk of exposure and understanding disease prevalence in real time.

## Supplemental Materials

10.4269/ajtmh.25-0452 Supplemental Materials

## References

[1] The Lancet, 2020. Zoonoses: Beyond the human–animal–environment interface. Lancet 396(10243): 1. 32622381 10.1016/S0140-6736(20)31486-0

[2] Rahman MT Sobur MA Islam MS Ievy S Hossain MJ El Zowalaty ME Rahman AT Ashour HM, 2020. Zoonotic diseases: Etiology, impact, and control. Microorganisms 8(9): 1405. 32932606 10.3390/microorganisms8091405 PMC7563794

[3] Jones KE Patel NG Levy MA Storeygard A Balk D Gittleman JL Daszak P, 2008. Global trends in emerging infectious diseases. Nature 451(7181): 990–993. 18288193 10.1038/nature06536 PMC5960580

[4] Simpson S Kaufmann MC Glozman V Chakrabarti A, 2020. Disease X: Accelerating the development of medical countermeasures for the next pandemic. Lancet Infect Dis 20(5): e108–e115. 32197097 10.1016/S1473-3099(20)30123-7 PMC7158580

[5] Ezenwa VO, , 2015. Interdisciplinarity and infectious diseases: An Ebola case study. PLoS Pathog 11(8): e1004992. 26247831 10.1371/journal.ppat.1004992 PMC4527690

[6] Lurie N Saville M Hatchett R Halton J, 2020. Developing Covid-19 vaccines at pandemic speed. N Engl J Med 382(21): 1969–1973. 32227757 10.1056/NEJMp2005630

[7] Weissleder R Lee H Ko J Pittet MJ, 2020. COVID-19 diagnostics in context. Sci Transl Med 12(546): eabc1931. 32493791 10.1126/scitranslmed.abc1931

[8] Worsley-Tonks KEL, , 2022. Strengthening global health security by improving disease surveillance in remote rural areas of low-income and middle-income countries. Lancet Glob Health 10(4): e579–e584. 35303467 10.1016/S2214-109X(22)00031-6 PMC8923676

[9] Schoepp RJ Rossi CA Khan SH Goba A Fair JN, 2014. Undiagnosed acute viral febrile illnesses, Sierra Leone. Emerg Infect Dis 20(7): 1176–1182. 24959946 10.3201/eid2007.131265 PMC4073864

[10] Grove JN, , 2011. Capacity building permitting comprehensive monitoring of a severe case of Lassa hemorrhagic fever in Sierra Leone with a positive outcome: Case report. Virol J 8: 314. 21689444 10.1186/1743-422X-8-314 PMC3283910

[11] Latinne A, , 2023. One Health surveillance highlights circulation of viruses with zoonotic potential in bats, pigs, and humans in Viet Nam. Viruses 15(3): 790. 36992498 10.3390/v15030790 PMC10053906

[12] Suu-Ire RD Obodai E Bonney JHK Bel-Nono SO Ampofo W Kelly TR, 2021. Viral zoonoses of national importance in Ghana: Advancements and opportunities for enhancing capacities for early detection and response. J Trop Med 2021: 8938530. 33574853 10.1155/2021/8938530 PMC7860970

[13] Mediannikov O Diatta G Fenollar F Sokhna C Trape J-F Raoult D, 2010. Tick-borne rickettsioses, neglected emerging diseases in rural Senegal. PLoS Negl Trop Dis 4(9): e821. 20856858 10.1371/journal.pntd.0000821 PMC2939048

[14] Clements TL Rossi CA Irish AK Kibuuka H Eller LA Robb ML Kataaha P Michael NL Hensley LE Schoepp RJ, 2019. Chikungunya and O’nyong-nyong viruses in Uganda: Implications for diagnostics. Open Forum Infect Dis 6(3): ofz001. 31660384 10.1093/ofid/ofz001 PMC6411207

[15] Addo SO, , 2023. First molecular identification of multiple tick-borne pathogens in livestock within Kassena-Nankana, Ghana. Anim Dis 3: 1.

[16] Addo SO, , 2024. Molecular identification of Crimean–Congo haemorrhagic fever virus in *Hyalomma rufipes* and *Amblyomma variegatum* in the Upper East Region of Ghana. Arch Virol 169(3): 62. 38446223 10.1007/s00705-024-05983-y

[17] Addo SO, , 2023. Occurrence of *Rickettsia* spp. and *Coxiella burnetii* in ixodid ticks in Kassena-Nankana, Ghana. Exp Appl Acarol 90(1–2): 137–153. 37322233 10.1007/s10493-023-00808-0

[18] Johnson SAM Asmah R Awuni JA Tasiame W Mensah GI Paweska JT Weyer J Hellferscee O Thompson PN, 2023. Evidence of Rift Valley fever virus circulation in livestock and herders in southern Ghana. Viruses 15(6): 1346. 37376647 10.3390/v15061346 PMC10300769

[19] Akuffo R, , 2016. Crimean–Congo hemorrhagic fever virus in livestock ticks and animal handler seroprevalence at an abattoir in Ghana. BMC Infect Dis 16: 324–325. 27392037 10.1186/s12879-016-1660-6 PMC4939019

[20] Bentil RE, , 2023. First whole genome sequencing of Crimean–Congo hemorrhagic fever virus (CCHFV) in tick species within Ghana. Transbound Emerg Dis 2023: 2063317. 40303792 10.1155/2023/2063317 PMC12016971

[21] Cohen CA, , 2025. A longitudinal analysis of memory immune responses in convalescent Crimean–Congo hemorrhagic fever survivors in Uganda. J Infect Dis 231(3): 762–772. 39248523 10.1093/infdis/jiae395

[22] Maze EA, , 2024. Serological cross-reactivity between Crimean–Congo haemorrhagic fever virus and Nairobi sheep disease virus glycoprotein C. Front Immunol 15: 1423474. 39902044 10.3389/fimmu.2024.1423474 PMC11788166

[23] Hayman DTS Yu M Crameri G Wang LF Suu-Ire R Wood JLN Cunningham AA, 2012. Ebola virus antibodies in fruit bats, Ghana, West Africa. Emerg Infect Dis 18(7): 1207–1209. 22710257 10.3201/eid1807.111654 PMC3376795

[24] Ogawa H Ohya K Ayizanga R Miyamoto H Shigeno A Yamada M Takashima Y Inoue-Murayama M Takada A Baboreka Kayang B, 2022. Detection of anti-ebolavirus antibodies in Ghanaian pigs. J Vet Med Sci 84(11): 1491–1494. 36123040 10.1292/jvms.22-0186 PMC9705824

[25] Bonney JK, , 2023. Marburg virus disease in Ghana. N Engl J Med 388(25): 2393–2394. 37342928 10.1056/NEJMc2300867

[26] Dzotsi EK, , 2012. The first cases of Lassa fever in Ghana. Ghana Med J 46(3): 166–170. 23661832 PMC3645162

[27] World Health Organization, 2023. *Weekly Bulletin on Outbreaks and Other Emergencies*. Week 10: 27 February to 5 March 2023. Available at: https://iris.who.int/server/api/core/bitstreams/17dffd57-a1e5-4fc0-859e-009ae4155b5a/content. Accessed March 26, 2026.

[28] Agboli E Tomazatos A Maiga-Ascofaré O May J Lühken R Schmidt-Chanasit J Jöst H, 2022. Arbovirus epidemiology: The mystery of unnoticed epidemics in Ghana, West Africa. Microorganisms 10(10): 1914. 36296190 10.3390/microorganisms10101914 PMC9610185

[29] Chan KR Ismail AA Thergarajan G Raju CS Yam HC Rishya M Sekaran SD, 2022. Serological cross-reactivity among common flaviviruses. Front Cell Infect Microbiol 12: 975398. 36189346 10.3389/fcimb.2022.975398 PMC9519894

[30] Morales-Núñez JJ Muñoz-Valle JF Torres-Hernández PC Hernández-Bello J, 2021. Overview of neutralizing antibodies and their potential in COVID-19. Vaccines (Basel) 9(12): 1376. 34960121 10.3390/vaccines9121376 PMC8706198

