# Supplementary material for: Cross-Sectional Analysis of Serologic Response to Arthropod-Borne and Hemorrhagic Fever Viruses in Ghanaian Livestock Herders
Source: Am J Trop Med Hyg. 2026 May 5;115(1):145–52. doi: 10.4269/ajtmh.25-0452 (PMC13326926; doi:10.4269/ajtmh.25-0452)
Supplement: Supplemental Materials [file tpmd250452.SD1.pdf]

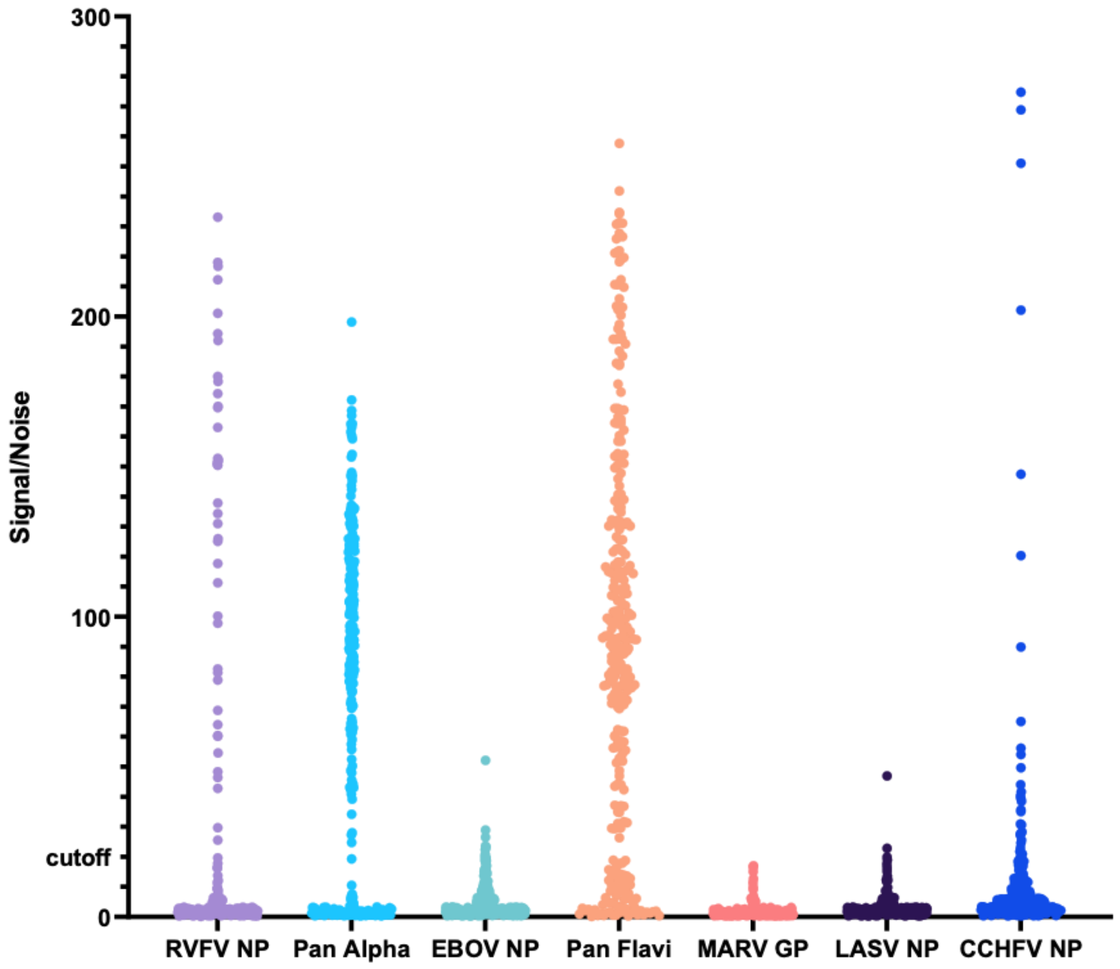

Figure S1: Magpix results showing a scatter plot of Signal-to-Noise values for viral targets.

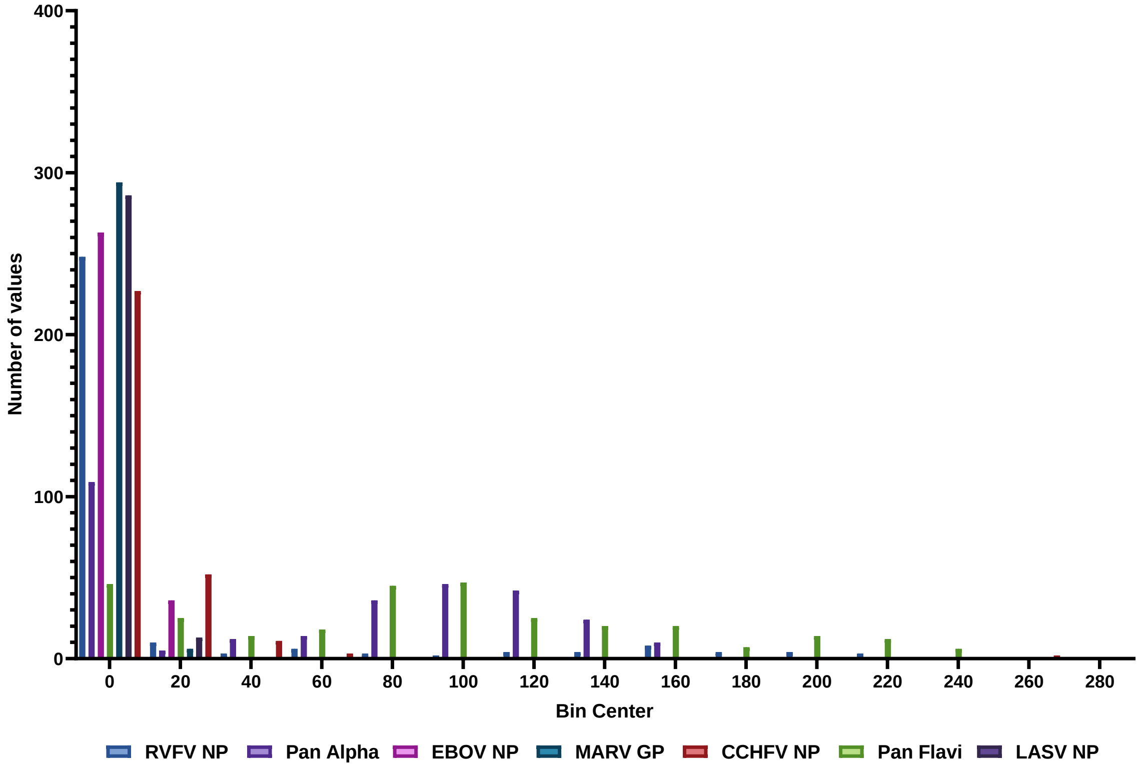

Figure S2: Signal-to-Noise frequency distribution of viral targets.

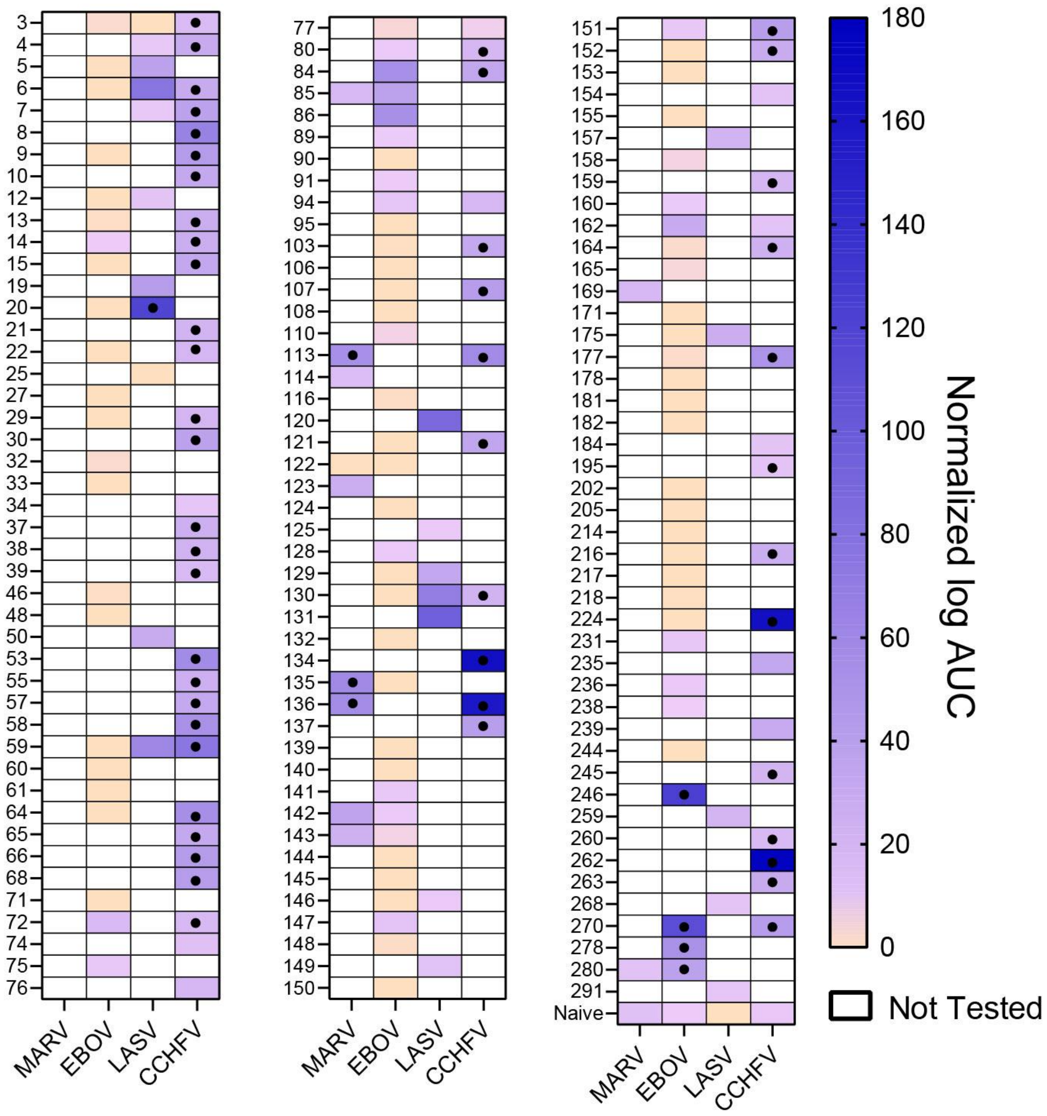

Figure S3: BSL4 Virus microneutralization results shown as a heat map of normalized log area under the curve (AUC). Dots represent positive samples relative to logAUC of naïve samples.

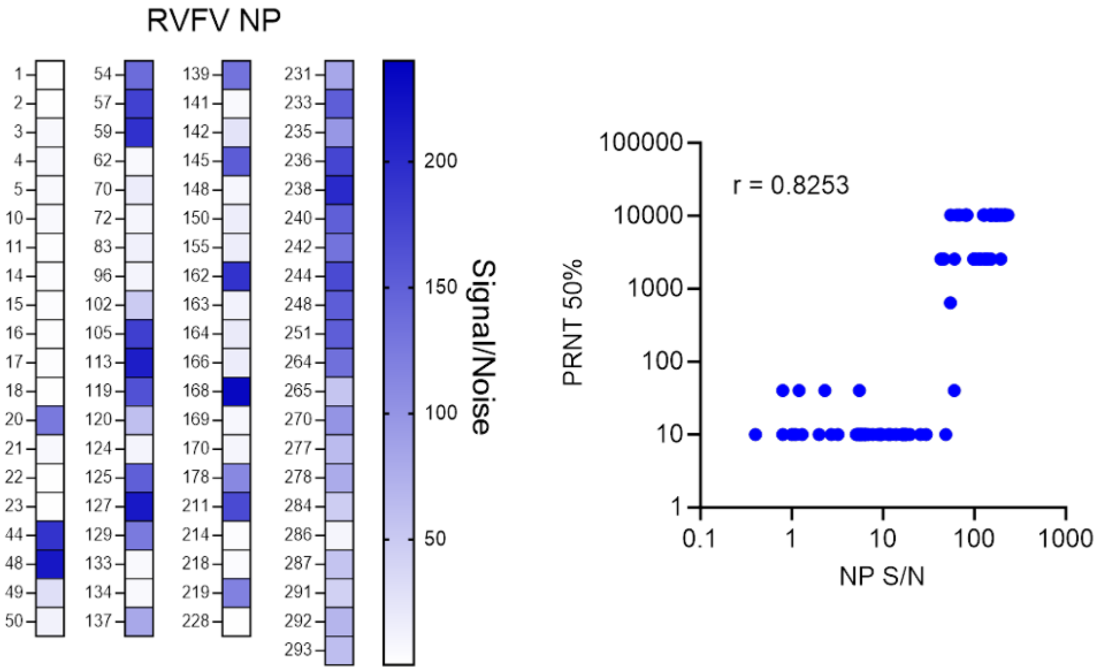

Figure S4: RVFV Magpix results shown as a heatmap of signal/noise (S/N) values from selected samples tested by PRNT (left graph). Correlation of MP12 PRNT50 and S/N from RVFV Magpix assay (right graph).   PRNT50 values are in reciprocal plasma dilutions.

Table S1: List of recombinant antigens, antigen source, and bead regions for multiplex immunoassay

Agent	Antigen	Source	Bead Region
Pan Flavi	VLP	Native Antigen Company	30
Ebola (EBOV)	NP	University of Hawaii	66
Pan Alpha	VLP	Native Antigen Company	25
CCHF (CCHFV)	NP	USAMRIID	78
Lassa (LASV)	NP	University of Hawaii	75
Marburg (MARV)	GP	Native Antigen Company	55
Rift Valley (RVFV)	NP	Native Antigen Company	22

Table S2a: Association with animal activities prior to the study and seroprevalence of infection

Characteristics	N(%)	Pan Alpha			Pan Flavi			RVFV		
		n(%)	95%CI	*p-value*[a]	n(%)	95%CI	*p-value*[a]	n(%)	95%CI	*p-value*[b]
**Animal cared for within a year**										
Live cattle										
Yes	183(61.0)	124(67.8)	60.6 - 74.2	0.033*	155(84.70)	78.7 - 89.2	<0.001*	23(12.6)	8.5 - 18.2	0.242
No	177(29.0)	65(55.6)	46.4 - 64.4		78(66.7)	57.6 - 74.7		21(18.0)	12.0 - 26.0	
Cattle parts										
Yes	122(40.7)	73(59.8)	50.8 - 68.2	0.394	81(66.4)	57.5 - 74.3	<0.001*	26(21.3)	14.9 - 29.5	0.008*
No	178(59.3)	116(65.2)	57.8 - 71.8		152(85.4)	79.4 - 89.9		18(10.1)	6.4 - 15.5	
Live sheep										
Yes	104(34.7)	78(75.0)	65.7 - 82.4	0.002*	95(91.4)	84.1 - 95.5	<0.001*	10(9.6)	5.2 - 17.0	0.086
No	196(65.3)	111(56.6)	49.6 - 63.4		138(70.4)	63.6 - 76.4		34(17.4)	12.6 - 23.3	
Sheep parts										
Yes	49(16.3)	38(77.6)	63.6 - 87.2	0.021*	43(87.8)	75.0 - 94.5	0.09	7(14.3)	6.9 - 27.3	1.000
No	251(83.7)	151(60.2)	53.9 - 66.1		190(75.7)	70.0 - 80.6		37(14.7)	10.9 - 19.7	
Live goat										
Yes	137(45.7)	94(68.6)	60.3 - 75.9	0.065	117(85.4)	78.4 - 90.4	0.003*	11(8.0)	4.5 - 14.0	0.003*
No	163(54.3)	95(58.3)	50.5 - 65.7		116(71.2)	63.7 - 77.6		33(20.3)	14.7 - 27.2	
Goat parts										
Yes	49(16.3)	37(75.5)	61.4 - 85.7	0.053	46(93.9)	82.4 - 98.0	0.002*	6(12.2)	5.5 - 24.9	0.825
No	251(83.7)	152(60.6)	54.3 - 66.5		187(74.5)	68.7 - 79.5		38(15.1)	11.1 - 20.2	
Live pig										
Yes	24(8.0)	14(58.3)	37.9 - 76.3	0.622	20(83.3)	62.4 - 93.8	0.615	2(8.3)	2.0 - 28.6	0.549
No	276(92.0)	175(63.4)	57.5 - 68.9		213(77.2)	71.8 - 81.8		42(15.2)	11.4 - 20.0	
Pig parts										
Yes	10(3.3)	4(40.0)	14.9 - 71.8	0.125	6(60.0)	28.2 - 85.1	0.238	1(10.0)	1.2 - 49.7	1.000
No	290(96.7)	185(63.8)	58.1 - 69.2		227(78.3)	73.1 - 82.7		43(14.8)	11.2 - 19.4	
**Total**	**300**	**189(63.0)**	**57.4 - 68.3**		**233(77.7)**	**72.6 - 82.0**		**44(14.7)**	**11.1 - 19.2**	

[a]*p-value* was obtained using chi-square test; [b]p-value was obtained using fisher's exact test; *Statistical significance at $p<0.05$

Table S2b: Association with animal activities prior to the study and seroprevalence of infection

Characteristics	N(%)	EBOV			CCHFV		
		n(%)	95%CI	*p-value*[b]	n(%)	95%CI	*p-value*[b]
**Animal cared for within a year**							
Live cattle							
Yes	183(61.0)	6(3.3)	1.4 - 7.1	0.253	15(8.0)	5.0 - 13.2	0.361
No	177(29.0)	1(0.9)	0.1 - 5.9		5(5.1)	2.3 - 11.0	
Cattle parts							
Yes	122(40.7)	0(0.0)	-	0.044*	3(2.5)	0.8 - 7.4	0.011*
No	178(59.3)	7(3.9)	1.9 - 8.1		18(10.1)	6.4 - 15.5	
Live sheep							
Yes	104(34.7)	4(3.9)	1.4 - 9.9	0.241	7(6.7)	3.2 - 13.5	1.000
No	196(65.3)	3(1.5)	0.5 - 4.7		14(7.1)	4.2 - 11.7	
Sheep parts							
Yes	49(16.3)	1(2.0)	0.3 - 13.4	1.000	1(2.0)	0.3 - 13.4	0.218
No	251(83.7)	6(2.4)	1.1 - 5.2		20(8.0)	5.2 - 12.1	
Live goat							
Yes	137(45.7)	5(3.6)	1.5 - 8.5	0.252	10(7.3)	4.0 - 13.1	1.000
No	163(54.3)	2(1.2)	0.3 - 4.8		11(6.8)	3.8 - 11.8	
Goat parts							
Yes	49(16.3)	1(2.0)	0.3 - 13.4		4(8.2)	3.1 - 20.1	0.759
No	251(83.7)	6(2.4)	1.1 - 5.2	1.000	17(6.8)	4.2 - 10.7	
Live pig							
Yes	24(8.0)	3(12.5)	4.0 - 33.1	0.013*	2(8.3)	2.0 - 28.6	0.679
No	276(92.0)	4(1.5)	0.5 - 3.8		19(6.9)	4.4 - 10.6	
Pig parts							
Yes	10(3.3)	1(10.0)	1.2 - 49.7	0.213	0(0.0)	-	1.000
No	290(96.7)	6(2.1)	0.9 - 4.5		21(7.2)	4.8 - 10.9	
**Total**	**300**	**7(2.3)**	**1.1 - 4.8**		**21(7.0)**	**4.6 - 10.5**	

[a]*p-value* was obtained using chi-square test; [b]p-value was obtained using fisher's exact test; *Statistical significance at $p<0.05$

Table S3a: Association with animal activities prior to the study and seroprevalence of infection

Characteristics	N(%)	Pan Alpha			Pan Flavi			RVFV		
		n(%)	95%CI	*p-value*[a]	n(%)	95%CI	*p-value*[a]	n(%)	95%CI	*p-value*[b]
**Activity involved in the last 3months**										
Care for live animals										
Yes	252(84.0)	164(65.1)	59.0 - 70.7	0.87	207(82.1)	76.9 - 86.4	<0.001*	27(10.7)	7.4 - 15.2	<0.001*
No	48(16.0)	25(52.1)	38.0 - 62.0		26(54.2)	40.0 - 67.8		17(35.4)	23.1 - 50.0	
Cut animal's throat										
Yes	120(40.0)	73(60.8)	51.8 - 69.2	0.526	98(81.7)	73.6 - 87.7	0.174	21(17.50)	11.7 - 25.4	0.318
No	180(60.0)	116(64.4)	57.1 - 71.1		135(75.0)	68.1 - 80.8		23(12.8)	8.6 - 18.5	
Skinned animals										
Yes	75(25.0)	50(66.7)	55.2 - 76.5	0.448	59(78.7)	67.8 - 86.7	0.81	19(25.3)	16.7 - 36.5	0.004*
No	225(75.0)	139(61.8)	55.2 - 67.9		174(77.3)	71.4 - 82.4		25(11.1)	7.6 - 36.5	
Collected animal blood										
Yes	46(15.4)	33(71.7)	57.0 - 83.0	0.192	40(87.0)	73.6 - 94.1	0.108	9(19.6)	10.4 - 33.8	0.276
No	253(84.6)	156(61.7)	55.5 - 67.5		193(76.3)	70.6 - 81.2		34(13.4)	9.7 - 18.3	
Handled animal parts										
Yes	191(63.7)	116(60.7)	53.6 - 67.4	0.282	141(73.8)	67.1 - 79.6	0.034*	30(15.7)	11.1 - 21.6	0.611
No	109(36.3)	73(67.0)	57.5 - 75.2		92(84.4)	76.3 - 90.1		14(12.8)	7.7 - 20.6	
Butchered carcasses										
Yes	87(29.0)	54(62.1)	51.4 - 71.7	0.831	67(77.0)	66.9 - 84.7	0.862	16(18.4)	11.5 - 28.0	0.281
No	213(71.0)	135(63.4)	56.7 - 69.6		166(77.9)	71.8 - 83.0		28(13.2)	9.2 - 18.4	
Animal Clean up										
Yes	103(34.3)	64(62.1)	52.3 - 71.0	0.832	87(84.5)	76.0 - 90.3	0.041*	12(11.7)	6.7 - 19.5	0.308
No	197(65.7)	125(63.5)	56.5 - 69.9		146(74.1)	67.5 - 79.8		32(16.2)	11.7 - 22.1	
**Animal cared for outside of work**										
Yes	153(51.0)	102(66.7)	58.8 - 73.7	0.18	124(81.1)	74.0 - 86.5	0.152	18(11.8)	7.5 - 18.0	0.191
No	147(49.0)	87(59.2)	51.0 - 66.9		109(74.2)	66.4 - 80.6		26(17.7)	12.3 - 24.8	
**Total**	**300**	**189(63.0)**	**57.4 - 68.3**		**233(77.7)**	**72.6 - 82.0**		**44(14.7)**	**11.1 - 19.2**	

[a]*p-value* was obtained using chi-square test; [b]p-value was obtained using fisher's exact test; *Statistical significance at $p<0.05$

Table S3b: Association with animal activities prior to the study and seroprevalence of infection

Characteristics	N(%)	EBOV			CCHFV		
		n(%)	95%CI	*p-value*[b]	n(%)	95%CI	*p-value*[b]
**Animal cared for within a year**							
Live cattle							
Yes	183(61.0)	7(2.8)	1.2 - 5.7	0.602	20(7.9)	5.2 - 12.0	0.218
No	177(29.0)	0(0.0)	-		1(2.1)	0.3 - 13.7	
Cattle parts							
Yes	122(40.7)	4(3.3)	1.2 - 8.6	0.443	6(5.0)	2.2 - 10.7	0.357
No	178(59.3)	3(1.7)	0.5 - 5.1		15(8.3)	5.0 - 13.4	
Live sheep							
Yes	104(34.7)	2(2.7)	0.7 - 10.2	1.000	4(5.3)	2.0 - 13.5	0.611
No	196(65.3)	5(2.2)			17(7.6)	4.7 - 11.8	
Sheep parts							
Yes	49(16.3)	3(6.5)	2.1 - 18.6	0.076	3(6.5)	2.1 - 18.6	1.000
No	251(83.7)	4(1.6)	0.6 - 4.1		18(7.1)	4.5 - 11.0	
Live goat							
Yes	137(45.7)	7(3.7)	1.7 - 7.5	0.051	9(4.7)	2.5 - 8.8	0.058
No	163(54.3)	0(0.0)	-		12(11.0)	6.3 - 18.5	
Goat parts							
Yes	49(16.3)	2(2.3)	0.6 - 8.8	1.000	3(3.5)	1.1 - 10.3	0.142
No	251(83.7)	5(2.4)	1.0 - 5.5		18(8.5)	5.4 - 13.0	
Live pig							
Yes	24(8.0)	4(3.9)	1.4 - 10.0		3(2.9)	0.9 - 8.7	0.056
No	276(92.0)	3(1.5)	0.4 - 4.6	0.237	18(9.1)	5.8 - 14.1	
Pig parts							
Yes	10(3.3)	5(3.3)	1.3 - 7.7	0.448	12(7.8)	4.5 - 13.4	0.653
No	290(96.7)	2(1.4)	0.3 - 5.3		9(6.1)	3.3 - 11.4	
**Total**	**300**	**7(2.3)**	**1.1 - 4.8**		**21(7.0)**	**4.6 - 10.5**	

[a]*p-value* was obtained using chi-square test; [b]p-value was obtained using fisher's exact test; *Statistical significance at $p<0.05$

Table S4a: Association with type of animal cared for outside work and seroprevalence of infection

Characteristics	N(%)	Pan Alpha			Pan Flavi			RVFV		
		n(%)	95%CI	*p-value*[a]	n(%)	95%CI	*p-value*[a]	n(%)	95%CI	*p-value*[b]
**Animal type cared for, n=153**										
Cattle										
Yes	66(43.1)	44(66.7)	54.3 - 77.1	1.000	53(80.3)	68.8 - 88.3	0.838	8(12.1)	6.1 - 22.6	1.000
No	87(56.9)	58(66.7)	56.0 - 75.9		71(81.6)	71.9 - 88.5		10(11.5)	6.2 - 20.2	
Sheep										
Yes	67(43.8)	52(77.6)	65.9 - 86.1	0.011*	61(91.0)	81.3 - 96.0	0.005*	9(13.4)	7.1 - 24.0	0.619
No	86(56.2)	50(58.1)	47.4 - 68.2		63(73.3)	62.8 - 81.6		9(10.5)	5.5 - 19.1	
Goat										
Yes	91(59.5)	62(68.1)	57.8 - 77.0	0.641	76(83.5)	74.3 - 89.9	0.345	12(13.2)	7.6 - 21.9	0.614
No	62(40.5)	40(64.5)	51.7 - 75.5		48(77.4)	65.2 - 86.2		6(9.7)	4.4 - 20.1	
Pig										
Yes	27(17.6)	22(81.5)	61.9 - 92.3	0.072	26(96.30)	77.0 - 99.5	0.029*	4(14.8)	5.5 - 34.1	0.526
No	126(82.4)	80(63.5)	54.6 - 71.5		98(77.8)	69.6 - 84.3		14(11.1)	6.7 - 18.0	
Chicken										
Yes	86(56.2)	64(74.4)	64.0 - 82.6	0.021*	74(86.1)	76.9 - 92.0	0.096	11(12.8)	7.2 - 21.8	0.802
No	67(43.8)	38(56.1)	44.5 - 68.2		50(74.5)	62.7 - 83.7		7(10.5)	5.0 - 20.5	
Pigeons										
Yes	5(3.3)	4(80.0)	25.3 - 97.9	0.665	5(100)	-	0.584	1(20.0)	2.1 - 74.7	0.47
No	119(96.7)	98(66.2)	58.1 - 73.4		119(80.4)	73.1 - 86.1		17(11.5)	7.2 - 17.8	
Bat										
Yes	3(2.0)	3(100)	-	0.551	3(100)	-	1.000	1(33.3)	2.5 - 90.6	0.315
No	150(98.0)	99(66.0)	58.0 - 73.2		121(80.7)	73.5 - 86.3		17(11.3)	7.1 - 17.6	

[a]*p-value* was obtained using chi-square test; [b]p-value was obtained using fisher’s exact test; *Statistical significance at $p<0.05$

Table S4b: Association with type of animal cared for outside work and seroprevalence of infection

Characteristics	N(%)	EBOV			CCHFV		
		**n(%)**	**95%CI**	***p-value*[b]**	**n(%)**	**95%CI**	***p-value*[b]**
**Animal type cared for, n=153**							
Cattle							
Yes	66(43.1)	3(4.6)	1.4 - 13.4	0.652	6(9.1)	4.1 - 19.0	0.763
No	87(56.9)	2(2.3)	0.6 - 8.9		6(6.9)	3.1 - 14.7	
Sheep							
Yes	67(43.8)	3(4.5)	1.4 - 13.2	0.654	5(7.5)	3.1 - 16.9	1.000
No	86(56.2)	2(2.3)	0.6 - 8.9		7(8.1)	3.9 - 16.2	
Goat							
Yes	91(59.5)	4(4.4)	1.6 - 11.3	0.649	8(8.8)	4.4 - 16.7	0.763
No	62(40.5)	1(1.6)			4(6.5)	2.4 - 16.2	
Pig							
Yes	27(17.6)	2(7.4)	1.8 - 26.0	0.213	3(11.1)	3.5 - 30.0	0.445
No	126(82.4)	3(2.4)	0.8 - 7.2		9(7.1)	3.7 - 13.3	
Chicken							
Yes	86(56.2)	4(4.7)	1.7 - 11.9	0.386	6(7.0)	3.1 - 14.8	0.765
No	67(43.8)	1(1.5)	0.2 - 10.1		6(9.0)	4.0 - 18.7	
Pigeons							
Yes	5(3.3)	1(20.0)	2.0 - 74.7	0.155	1(20.0)	2.1 - 74.7	0.339
No	119(96.7)	4(2.7)	1.0 - 7.0		11(7.4)	4.1 - 13.00	
Bat							
Yes	3(2.0)	1(33.3)	2.5 - 90.6	0.095	0(0.0)	-	1.000
No	150(98.0)	4(2.7)	9.9 - 7.0		12(8.0)	4.6 - 13.6	

[a]*p-value* was obtained using chi-square test; [b]p-value was obtained using fisher's exact test; *Statistical significance at $p<0.05$

Table S5a: Association with reported symptoms and seroprevalence of infection

Characteristics	N(%)	Pan Alpha			Pan Flavi			RVFV		
		n(%)	95%CI	*p-value*[a]	n(%)	95%CI	*p-value*[a]	n(%)	95%CI	*p-value*[b]
**Symptoms**										
Fever										
Yes	104(34.7)	77(74.0)	64.6 - 81.6	0.004*	91(87.5)	79.6 - 92.6	0.364	18(17.3)	11.1 - 25.9	0.242
No	196(65.3)	112(57.1)	50.0 - 63.9		142(72.5)	65.7 - 78.3		26(13.3)	9.2 - 18.8	
Headache										
Yes	149(49.7)	101(67.8)	59.8 - 74.8	0.088	119(79.9)	72.6 - 85.6	0.003*	20(13.4)	8.8 - 19.9	0.392
No	151(50.3)	88(58.3)	50.2 - 65.9		114(75.5)	68.0 - 81.7		24(15.9)	10.9 - 22.7	
Malaise										
Yes	135(45.0)	91(67.4)	59.0 - 74.8	0.153	112(83.0)	75.6 - 88.4	0.046*	21(15.6)	10.3 - 22.7	0.744
No	165(55.0)	98(59.4)	51.7 - 66.7		121(73.3)	66.0 - 79.6		23(13.9)	9.4 - 20.2	
Chills										
Yes	73(24.3)	50(68.5)	56.9 - 78.2	0.264	60(82.2)	71.6 - 89.4	0.286	11(15.1)	8.5 - 25.3	1.000
No	227(75.7)	139(61.2)	54.7 - 67.4		173(76.2)	70.2 - 81.3		33(14.5)	10.5 - 19.8	
Cough										
Yes	67(22.3)	44(65.7)	53.5 - 76.1	0.607	59(88.1)	77.7 - 94.0	0.020*	13(19.4)	11.5 - 30.8	0.24
No	233(77.7)	145(62.2)	55.6 - 68.3		174(74.7)	68.6 - 79.9		31(13.3)	9.4 - 18.3	
Sore throat										
Yes	52(17.3)	31(59.6)	45.7 - 72.1	0.578	39(75.0)	61.4 - 85.0	0.612	7(13.5)	6.5 - 25.9	1.000
No	248(82.7)	158(63.7)	57.5 - 69.5		194(78.2)	72.6 - 83.0		37(14.9)	11.0 - 19.9	
**Total**	**300**	**189(63.0)**	**57.4 - 68.3**		**233(77.7)**	**72.6 - 82.0**		**44(14.7)**	**11.1 - 19.2**	

[a]*p-value* was obtained using chi-square test; [b]p-value was obtained using fisher's exact test; *Statistical significance at $p<0.05$

Table S5b: Association with reported symptoms and seroprevalence of infection

Characteristics	N(%)	Pan Alpha			Pan Flavi			RVFV		
		n(%)	95%CI	*p-value*[a]	n(%)	95%CI	*p-value*[a]	n(%)	95%CI	*p-value*[b]
**Symptoms**										
Rash										
Yes	20(6.7)	8(40.0)	21.0 - 62.6	0.028	16(80.0)	56.4 - 92.5	0.789	5(25.0)	10.5 - 48.6	0.190
No	279(93.3)	180(64.5)	58.7 - 69.9		216(77.4)	72.1 - 82.0		39(14.0)	10.4 - 18.6	
Back pain										
Yes	124(41.3)	80(64.5)	55.7 - 72.5	0.648	99(79.8)	71.8 - 86.0	0.448	21(16.9)	11.3 - 24.7	1.000
No	176(58.7)	109(61.9)	54.5 - 68.8		134(76.1)	69.2 - 81.9		23(13.1)	8.8 - 18.9	
Joint pain										
Yes	147(49.0)	98(66.7)	58.6 - 73.9	0.197	121(82.3)	75.2 - 87.7	0.058	21(14.3)	9.5 - 21.0	0.872
No	153(51.0)	91(59.5)	51.5 - 67.0		112(73.2)	65.6 - 79.7		23(15.0)	10.2 - 21.7	
Nausea										
Yes	49(16.3)	36(73.5)	59.3 - 84.0	0.097	40(81.6)	68.1 - 90.2	0.466	5(10.2)	4.3 - 22.5	0.387
No	251(83.7)	153(61.0)	54.7 - 66.8		193(76.9)	71.2 - 81.7		39(15.5)	11.5 - 20.6	
Vomiting										
Yes	24(8.0)	17(70.8)	49.6 - 85.7	0.407	19(79.2)	58.0 - 91.3	0.854	3(12.5)	4.0 - 33.1	1.000
No	276(92.0)	172(62.3)	56.4 - 67.9		214(77.5)	72.2 - 82.1		41(14.9)	11.1 - 19.6	
Diarrhea										
Yes	28(9.3)	21(75.0)	55.6 - 87.8	0.167	26(92.9)	74.9 - 98.3	0.043*	3(10.7)	3.4 - 29.0	0.779
No	272(90.7)	168(61.8)	55.8 - 67.4		207(76.1)	70.6 - 80.8		41(15.1)	11.3 - 19.9	
Weight loss										
Yes	64(21.3)	53(82.8)	71.4 - 90.3	< 0.001	57(89.1)	78.6 - 94.7	0.014*	10(15.6)	8.6 - 26.8	0.842
No	236(78.7)	136(57.6)	51.1 - 63.8		176(74.6)	68.6 - 79.8		34(14.4)	10.5 - 19.5	
**Total**	**300**	**189(63.0)**	**57.4 - 68.3**		**233(77.7)**	**72.6 - 82.0**		**44(14.7)**	**11.1 - 19.2**	

[a]*p-value* was obtained using chi-square test; [b]p-value was obtained using fisher's exact test; *Statistical significance at $p<0.05$

Table S5c: Association with reported symptoms and seroprevalence of infection

Characteristics	N(%)	EBOV			CCHFV		
		n(%)	95%CI	*p-value*[b]	n(%)	95%CI	*p-value*[b]
**Symptoms**							
Fever							
Yes	104(34.7)	5(4.8)	2.0 - 11.1	0.051	12(11.5)	4.1 - 12.9	0.032*
No	196(65.3)	2(1.0)	0.2 - 99.7		9(4.6)	3.6 - 11.9	
Headache							
Yes	149(49.7)	7(4.70)	2.2 - 9.6	0.007*	11(7.4)	6.6 - 19.3	0.825
No	151(50.3)	0(0.0)	-		10(6.6)	2.3 - 8.6	
Malaise							
Yes	135(45.0)	4(3.0)	1.1 - 7.7	0.705	11(8.2)	4.5 - 14.2	0.503
No	165(55.0)	3(1.8)	0.6 - 5.5		10(6.1)	3.3 - 10.9	
Chills							
Yes	73(24.3)	3(4.1)	1.3 - 12.1	0.367	7(9.6)	4.6 - 18.9	0.303
No	227(75.7)	4(1.8)	0.6 - 4.6		14(6.2)	3.7 - 10.2	
Cough							
Yes	67(22.3)	3(4.5)	1.4 - 13.1	0.188	8(11.9)	6.0 - 22.3	0.099
No	233(77.7)	4(1.7)	0.6 - 4.5		13(5.6)	3.2 - 9.4	
Sore throat							
Yes	52(17.3)	1(1.9)	0.3 - 12.7	1.000	3(5.8)	1.8 - 16.6	1.000
No	248(82.7)	6(2.4)	1.1 - 5.3		18(7.3)	4.6 - 11.3	
**Total**	**300**	**189(63.0)**	**57.4 - 68.3**		**233(77.7)**	**72.6 - 82.0**	

[a]*p-value* was obtained using chi-square test; [b]p-value was obtained using fisher’s exact test; *Statistical significance at $p<0.05$

Table S5d: Association with reported symptoms and seroprevalence of infection

Characteristics	N(%)	EBOV			CCHFV		
		n(%)	95%CI	*p-value*[b]	n(%)	95%CI	*p-value*[b]
**Symptoms**							
Rash							
Yes	20(6.7)	1(5.0)	0.6 - 29.5	0.387	4(20.0)	7.5 - 43.6	0.042
No	279(93.3)	6(2.2)	1.0 - 4.7		17(6.1)	3.8 - 9.6	
Back pain							
Yes	124(41.3)	4(3.2)	1.2 - 8.3	0.453	5(4.0)	1.7 - 9.4	0.11
No	176(58.7)	3(1.7)	0.5 - 5.2		16(9.1)	5.6 - 14.4	
Joint pain							
Yes	147(49.0)	2(1.4)	0.3 - 5.3	0.448	11(7.5)	4.2 - 13.1	0.823
No	153(51.0)	5(3.3)	1.4 - 7.7		10(6.5)	3.5 - 11.8	
Nausea							
Yes	49(16.3)	2(4.1)	1.0 - 15.2	0.321	3(6.1)	2.0 - 17.6	1.000
No	251(83.7)	5(2.0)	0.9 - 4.7		18(7.2)	4.6 - 11.1	
Vomiting							
Yes	24(8.0)	1(4.2)	0.6 - 25.3	0.446	2(8.3)	2.0 - 28.6	0.679
No	276(92.0)	6(2.2)	1.0 - 4.8		19(6.9)	4.4 - 10.6	
Diarrhea							
Yes	28(9.3)	0(0.0)	-	1.000	4(14.3)	5.3 - 33.0	0.119
No	272(90.7)	7(2.6)	1.2 - 5.3		17(6.3)	3.9 - 9.8	
Weight loss							
Yes	64(21.3)	1(1.6)	0.2 - 10.5	1.000	5(7.8)	3.2 - 17.6	0.784
No	236(78.7)	6(2.5)	1.1 - 5.6		16(6.8)	4.2 - 10.8	
**Total**	**300**	**189(63.0)**	**57.4 - 68.3**		**233(77.7)**	**72.6 - 82.0**	

[a]*p-value* was obtained using chi-square test; [b]p-value was obtained using fisher's exact test; *Statistical significance at $p<0.05$
